# The formation of a large summertime Saharan dust plume: Convective and synoptic-scale analysis

**DOI:** 10.1002/2013JD020667

**Published:** 2014-02-26

**Authors:** A J Roberts, P Knippertz

**Affiliations:** 1 School of Earth and Environment, University of LeedsLeeds, UK

**Keywords:** WRF, haboob, MCS, Sahara, dust, convection

## Abstract

Haboobs are dust storms produced by the spreading of evaporatively cooled air from thunderstorms over dusty surfaces and are a major dust uplift process in the Sahara. In this study observations, reanalysis, and a high-resolution simulation using the Weather Research and Forecasting model are used to analyze the multiscale dynamics which produced a long-lived (over 2 days) Saharan mesoscale convective system (MCS) and an unusually large haboob in June 2010. An upper level trough and wave on the subtropical jet 5 days prior to MCS initiation produce a precipitating tropical cloud plume associated with a disruption of the Saharan heat low and moistening of the central Sahara. The restrengthening Saharan heat low and a Mediterranean cold surge produce a convergent region over the Hoggar and Aïr Mountains, where small convective systems help further increase boundary layer moisture. Emerging from this region the MCS has intermittent triggering of new cells, but later favorable deep layer shear produces a mesoscale convective complex. The unusually large size of the resulting dust plume (over 1000 km long) is linked to the longevity and vigor of the MCS, an enhanced pressure gradient due to lee cyclogenesis near the Atlas Mountains, and shallow precipitating clouds along the northern edge of the cold pool. Dust uplift processes identified are (1) strong winds near the cold pool front, (2) enhanced nocturnal low-level jet within the aged cold pool, and (3) a bore formed by the cold pool front on the nocturnal boundary layer.

## Key Points

- Multiscale analysis of meteorology which formed a Saharan MCS and dust plume
- Successful WRF simulation of Saharan MCS and convectively generated cold pool
- Identification of dust uplift processes associated with convective cold pool

## 1 Introduction

Airborne Saharan mineral dust impacts on the Earth's climate system through direct and indirect radiative effects [*Goudie and Middleton*, 2001; *Slingo et al.*, 2006; *Goudie*, 2009; *Shao et al.*, 2011]. It acts as a fertilizer in oceanic regions low in nutrients and has also been suggested as a source of nutrients in terrestrial regions such as the Amazon [*Jickells et al.*, 2005; *Bristow et al.*, 2010; *Ohde and Siegel*, 2010]. Airborne dust also strongly influences human life through respiratory and eye problems, pathogenic transport, damage to agriculture, and increased risk for vehicular and aircraft traffic [*Sterk*, 2003; *Sultan et al.*, 2005; *Derbyshire*, 2007]. The dynamics of different dust deflation processes and their relative importance are poorly understood. The low population density and political instability of the Sahara mean that there are few synoptic stations and that it is difficult to make additional in situ observations. Therefore, satellite observations and modeling studies are useful tools for improving our understanding of dust lofting phenomena in the Sahara [*Knippertz and Todd*, 2012].

In the boreal summer the region with greatest dust loading shifts from the Bodélé Depression in Chad to the central western Sahara [*Washington et al.*, 2003; *Engelstaedter and Washington*, 2007; *Schepanski et al.*, 2007; *Knippertz and Todd*, 2010; *Fiedler et al.*, 2013]. This is linked to the onset of the West African monsoon (WAM) and a strengthening of the Saharan heat low. The heat low provides the pressure gradient needed for the production of low-level jets (LLJs), while the monsoon winds bring the moisture needed for the production of mesoscale convective systems (MCSs) [*Hamilton et al.*, 1945]. Some of the dust lifted in the western Sahara hot spot is raised by haboobs [*Knippertz and Todd*, 2010]. Haboobs occur when melting and evaporating hydrometeors in convective storms create downdrafts and cold pools [*Byers*, 1949]. The winds associated with the leading edge of a cold pool in a desert are strong enough to lift loose, dry surface sediments, and create imposing walls of dust, often over 1 km in height.

Convective haboobs have been documented in the Sahel and Sahara [*Sutton*, 1925; *Farquharson*, 1937; *Hamilton et al.*, 1945; *Freeman*, 1952; *Lawson*, 1971; *Flamant et al.*, 2007; *Reinfried et al.*, 2009; *Knippertz et al.*, 2009; *Emmel et al.*, 2010; *Solomos et al.*, 2012; *Roberts and Knippertz*, 2012], the Arabian Peninsula [*Membery*, 1985; *Miller et al.*, 2008], the Southern U.S. [*Brazel and Nickling*, 1986; *Chen and Fryrear*, 2002], and the Taklimakan and Gobi deserts in China [*Mitsuta et al.*, 1995; *Takemi*, 1999], and their presence in Australia is mentioned in *Strong et al.* [2011]. Although the relative importance of haboobs for dust uplift compared with other phenomena is not well known, recent work suggests that their activity during the WAM season maybe responsible for approximately 50% of deflation [*Marsham et al.*, 2008, 2011, 2013a; *Heinold et al.*, 2013]. Observations have also shown that African haboobs are responsible for considerable low-level moisture fluxes into the Sahara [*Knippertz et al.*, 2007; *Marsham et al.*, 2013a]. The lack of this effect in global models is a major source of bias in modeling the central Saharan heat low [*Garcia-Carreras et al.*, 2013; *Marsham et al.*, 2013b]. Therefore, a better understanding of haboobs will help improve modeling of the WAM system.

The mesoscale nature of the convective storms that are the source of haboobs means that usually they have front lengths on the order of tens to a hundred kilometers. However, in some circumstances they appear to be able to reach much larger sizes, with front lengths of more than 1000 km and traveling similar distances [*Miller et al.*, 2008; *Roberts and Knippertz*, 2012].

Northward surges of the WAM help to produce MCSs close enough to the dusty northern Sahel and Sahara to form haboobs. Factors that influence the behavior of the monsoon flow and produce northward excursions include westward propagating Rossby waves on the African easterly jet (AEJ) known as African easterly waves [*Berry and Thorncroft*, 2005; *Cuesta et al.*, 2010; *Knippertz and Todd*, 2010], interactions between the tropics and the midlatitudes [*Knippertz*, 2008; *Knippertz and Todd*, 2010], and feedback between mesoscale and synoptic-scale meteorology such as convective cold pool strengthening of the monsoonal flow [*Flamant et al.*, 2007; *Marsham et al.*, 2008; *Cuesta et al.*, 2010]. The mesoscale nature of some of these features, which have the ability to feed back onto the synoptic scale, implies that they can be poorly represented in coarse-resolution global products, creating disagreement between them. The sparse observational network means that reanalyses and operation analyses are poorly constrained. This makes limited-area modeling a difficult task in this region and means that the outcome of a simulation is largely dependent on the data used for initialization.

The majority of work conducted on haboobs has focused on observations. These are either case studies or statistical analyses of a number of events at a single site. Recent studies have simulated haboob events using high resolution models. *Takemi* [2005] used a 3-D nonhydrostatic cloud resolving model including a dust scheme and initialized an idealized simulation using observations from an event in the Chinese Gobi desert. *Knippertz et al.* [2009], *Reinfried et al.* [2009], and *Solomos et al.* [2012] all modeled the production of cold pools in the northwestern Sahara close to the Atlas Mountains. Both *Takemi*[2005] and *Solomos et al.* [2012] conclude that haboobs are an efficient mechanism for lifting dust and that they have the capability to transport dust away from the surface and into the air above the cold pool. *Reinfried et al.* [2009] found that cold pool production was strongly dependent on convective parameterizations with the most realistic simulations explicitly resolving convective processes. *Knippertz et al.* [2009] found that microphysics had a moderate impact on cold pool evolution but that the length scale for turbulent vertical mixing had a stronger influence.

**Figure 1:**
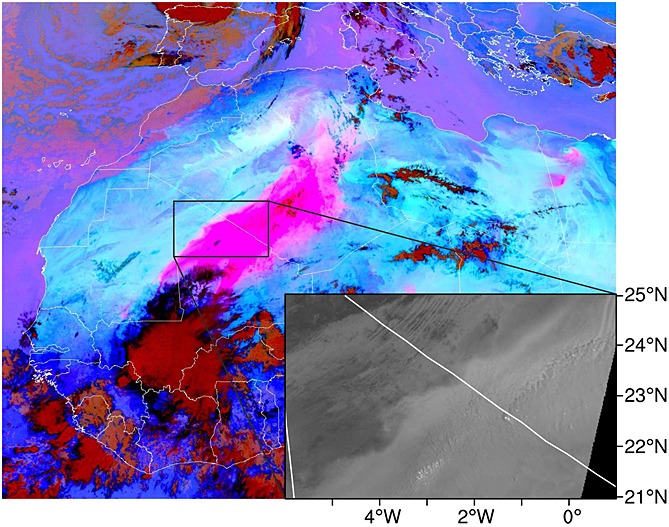
Spinning Enhanced Visual and Infrared Imager (SEVIRI) red, green, blue (RGB) dust image showing the extent of the large dust plume and the MCS that produced it at 1030 UTC 10 June 2010. The lifted dust detected is represented by magenta, deep clouds by red, midlevel clouds by orange, cirrus clouds by black, and the surface by blue. Inset is the Moderate Resolution Imaging Spectroradiometer (MODIS) band 4 (545–565 nm) visible radiance suggesting the production of horizontal shear instability along the edge of the old cold pool.

The focus of this study is the meteorology that produced an unusually large MCS and intense dust plume in the Sahara (Figure 1) and the dynamics of its associated cold pool. The case occurred between 8 and 10 June 2010 and was initiated over the Hoggar and Aïr Mountains in southern Algeria and northern Niger, respectively. The dust plume covered parts of Algeria, Mali, and Mauritania and was later deformed by the background flow and transported over the Mediterranean Sea. This paper (1) identifies the meteorological processes responsible for the production of the MCS, (2) tests whether the Weather Research and Forecasting (WRF) model is capable of simulating the case despite the data sparse nature of the region, and (3) examines the mesoscale dynamics to identify the processes important for dust deflation. To the best of our knowledge there have been no modeling studies that have looked into the production of haboobs in the central Sahara and none that focus on the generation of very large haboobs. Section 2 of this paper describes the observational and reanalysis data used, as well as the WRF model and its setup. Section 3 focuses on results from observational and reanalysis data, section 4 compares these findings with the WRF simulation of the case, and section 5 identifies potential dust uplift mechanisms from the simulation. Section 6 contains a summary and conclusions.

## 2 Data and Model Setup

### 2.1 Observational Data

Due to the sparse nature of surface and upper air stations in the region, satellite products are a key source of information. Products used include

1. EUMETSAT (European organization for the exploitation of meteorological satellites) Spinning Enhanced Visual and Infrared Imager (SEVIRI) red, green, blue (RGB) false color dust imagery. This product has a 15 min temporal resolution and a field of view covering the whole of Northern Africa. It uses a combination of brightness temperature (BT) and BT differences from SEVIRI channels 7, 9, and 10 to identify regions where dust has been raised. Several limitations have been identified including a strong dependence on column water vapor, lower tropospheric lapse rate, and the altitude of lifted dust [*Brindley et al.*, 2012].
2. North African Sand Storm Survey (NASCube) pseudo color visual and infrared imagery and temperature anomaly identification product. Derived from SEVIRI, this product also has a 15 min temporal resolution and a wide field of view. It uses two separate algorithms for the production of true and pseudo true color daytime and nighttime images using both visible and infrared SEVIRI channels. A cloud mask is generated using an artificial neural network, and anomalies related to lifted dust are identified from a 10 day cloud free average. Both SEVIRI-based products are excellent for tracking the development of systems that produce haboobs but only provide qualitative information on dust content.
3. Tropical Rainfall Measuring Mission (TRMM) 3B42 V7 rainfall retrievals are used to estimate rainfall in the region and to verify the WRF simulation. A combination of spaceborne radar, microwave, and infrared radiance data is used to create 3-hourly 0.25° × 0.25° resolution estimates of rainfall between 50°N and 50°S [*Huffman et al.*, 2007] (gauge data are used for calibration where available).
4. Moderate Resolution Imaging Spectroradiometer (MODIS) imagery of Level 1B calibrated and geolocated radiances are used to identify cloud and dust features. MODIS data provide a number of different channels in both visible and infrared wavelengths. However, with a maximum of four overpasses a day (from Terra and Aqua platforms) the use of MODIS data is limited and is used to illustrate important dynamic processes where possible.

Direct observations used are radiosonde launches from In Salah and Tamanrasset in Algeria, Agadez and Niamey in Niger, and Ouagadougou in Burkina-Faso.

### 2.2 ERA Interim Re-Analysis

To understand the large-scale meteorology associated with the generation of the MCS and the behavior of the subsequent dust plume, European Centre for Medium-Range Weather Forecasts (ECMWF) ERA Interim Re-Analysis is used [*Dee et al.*, 2011]. The reanalysis is achieved through 12 h four-dimensional variational assimilation (4D-Var) using the ECMWF operational forecast model (Cy31r2). It uses 60 vertical levels and has a T255 spectral resolution (approximately 79 km on Gaussian grid). The inability of the coarse-resolution forecast to accurately represent moist convective processes and the very sparse nature of in situ observations in the region mean that it was decided to only use ERA Interim data for the investigation of synoptic conditions, such as large-scale pressure gradients and the position and influence of the subtropical jets.

### 2.3 WRF Model Description and Setup

The WRF dynamics solver used is the advanced research WRF version 3.3.1. This includes fully compressible nonhydrostatic equations, terrain following vertical coordinates, Arakawa C-grid, second-order Runge-Kutta time integration, and both one- and two-way nesting options. A comprehensive description of the model architecture can be found in *Skamarock and Klemp*[2008].

The simulation was initialized at 0000 UTC on 7 June 2010 and used an external domain covering West Africa with 30 km grid spacing and 6-hourly boundary conditions provided by ECMWF operational analysis. Initially, three different global products were used to initialize the simulation (the National Centers for Environmental Prediction-Global Forecast System operational analysis, the ECMWF ERA Interim Re-Analysis, and the ECMWF operational analysis). However, only simulations initialized using ECMWF operational analysis generated deep convection in the right place and at the right time. This reemphasizes the point made in section 1 that global products are not always in agreement and suggests that a large factor in the success of a simulation is the choice of data used for its initialization.

**Figure 2:**
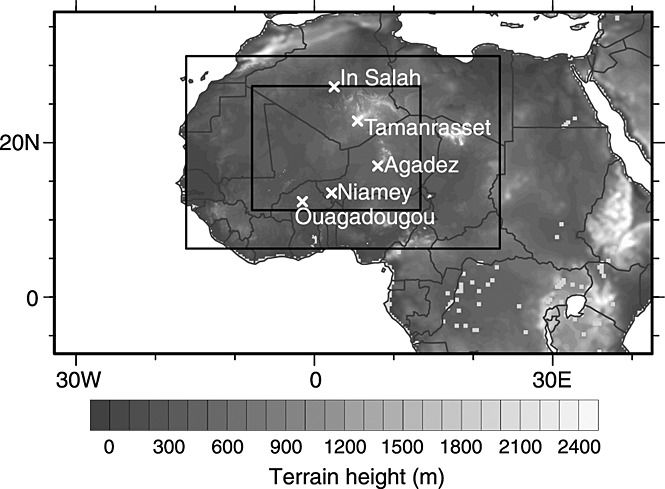
WRF domains with orography. Radiosonde stations used in the study are marked and named.

A three-tiered two-way nested structure was created. The intermediate domain has 10 km grid spacing and the internal domain 3.33 km grid (Figure 2). All domains have 50 vertical levels, with a higher concentration of levels in the lower atmosphere. The two outer domains rely on the Kain-Fritsch cumulus parameterization scheme, while the high-resolution inner domain explicitly resolves convection. This allows for better representation of convection and therefore the formation of mesoscale structures such as cold pools [*Reinfried et al.*, 2009]. The microphysics scheme used is the updated Thompson containing six hydrometeor classes including graupel and number concentrations for cloud ice and rain [*Thompson et al.*, 2008]. A number of sensitivity simulations were run with different combinations of planetary boundary layer scheme, land surface scheme, and surface layer scheme. From these simulations it was clear that the land surface scheme had a strong influence on the triggering of convective events, while the choice of planetary boundary layer scheme had a minor impact. The simulations using the Noah land surface model (a four-layer temperature and moisture scheme) produced deep convective clouds faster and more readily than the simpler five-layer thermal diffusion scheme. This meant that the initiation time for the MCS in simulations using the Noah land surface model were closer to that observed in satellite imagery. However, the subsequent behavior of the MCS was much more closely matched by the simulations using the five-layer thermal diffusion scheme. Therefore, the simulation shown here uses the five-layer thermal diffusion land surface scheme, the fifth generation Pennsylvania State University-NCAR mesoscale model (MM5) similarity surface layer scheme, and the Yonsei University, nonlocal-K scheme for the planetary boundary layer. Other physics options used include the rapid radiative transfer model for long-wave radiation and the Goddard two-stream multiband scheme for short-wave radiation. These settings closely resemble those used by the National Center for Atmospheric Research for hurricane forecasting [*Wang et al.*, 2012], suggesting their applicability to mesoscale convectively driven systems. This WRF simulation does not contain dust, and so conclusions about dust uplift are drawn from identifying meteorological processes capable of deflating surface dust. This is similar to the approach used by *Burton et al.* [2013].

It is noteworthy that the largest impact on simulation outcome was produced by varying the initialization data rather than changes to the model physics. This represents a significant problem for the high-resolution, limited area modeling community in data sparse regions. It confirms that there can be significant differences between products, which are ostensibly providing the best estimate of the atmosphere from the same (or very similar) observational data.

## 3 Observational Reanalysis

### 3.1 Synoptic-Scale Environment Prior to Triggering of Convection

**Figure 3:**
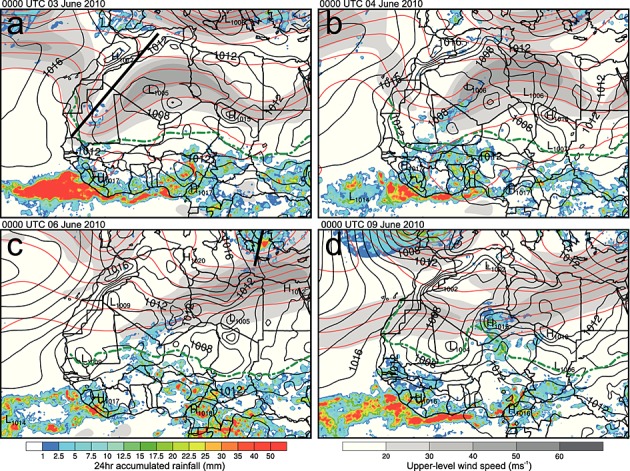
Synoptic-scale evolution before the initiation of the haboob producing MCS. Shown is the ERA Interim Re-Analysis 200 hPa geopotential (red lines), 200 hPa wind speed (gray shading), mean sea-level pressure in hectopascal (black isobars), 14°C 2 m dewpoint temperature over land showing the ITD (dashed green line) and the estimated 24 h accumulated rainfall from TRMM 3B42 V7 (color shading) at (a) 0000 UTC 3 June, (b) 0000 UTC 4 June, (c) 0000 UTC 06, and (d) 0000 UTC 9 June 2010. The thick black lines in Figures 3a and 3c highlight the axes of troughs discussed in the text.

Figure 3 shows the evolution of the synoptic-scale meteorology prior to the formation of the MCS. It includes 200 hPa geopotential and wind speed, mean sea-level pressure, and the position of the intertropical discontinuity (the surface interface between the moist WAM air to the south and the dry desert air to the north, hereafter ITD) from ERA Interim. Also shown is the TRMM 3B42 rainfall estimate for the 24 h proceeding the time indicated in Figures 3a–3d. On 3 June 2010, a week before the formation of the dust plume shown in Figure 1, a deep upper level trough is present with a southwest-northeast orientation from Senegal to northern Algeria (marked in Figure 3a). At this time the Saharan heat low can be seen as a 1005 hPa minimum in mean sea-level pressure over Algeria. The ITD is predominantly zonal (close to its climatological position) and there has been very light precipitation over the southern part of the trough in the proceeding 24 h (Figure 3a).

The wave on the subtropical jet propagates east, and by 4 June the southerly limb begins to weaken, producing a southwesterly subtropical jet streak over Mali and Algeria (Figure 3b). The heat low and ITD position remain largely unchanged at this time, but light rainfall is produced over a more widespread area (parts of Mauritania, Mali, and Algeria). This rainfall is coincident with the southern part of the jet streak and the synoptic-scale dynamics are similar to that of a tropical plume [*Fröhlich et al.*, 2013; *Knippertz and Martin*, 2005]. This suggests that near-surface or midlevel tropical air is advected north due to low-level convergence associated with the upper level flow.

By 6 June (Figure 3c) the upper level trough has started to decay and the jet straightened to be westerly, weakening the tropical plume. However, the cloudy/rainy conditions persist for 4 days and strongly influence the Saharan heat low, splitting it into two distinct regions, which can be seen in the isobars in Figure 3c. This is probably caused by the combination of reduced surface short-wave radiation and the production of a mesohigh associated with evaporating precipitation. Nevertheless, over this period (6 to 3 days before cold pool production) the ITD position remains largely zonal with little variation. Another important feature at this time is the development of a second trough over the eastern Mediterranean (marked in Figure 3c). The straightening of the subtropical jet and the positioning of this trough produces conditions similar to those examined by *Vizy and Cook* [2009]. Under this regime it was found that high surface pressure often develops over Libya and produces a Mediterranean cold air surge into the Sahara. The ERA Interim mean sea-level pressure develops a high which builds over the Mediterranean Sea and Tunisia and is later positioned over eastern Algeria and Libya.

Figure 3d shows the synoptic situation approximately 12 h after the initiation of the MCS. At this time the trough over the eastern Mediterranean is still present and the subtropical jet is largely westerly. The decay of the tropical plume after 6 June is followed by a reestablishment of the Saharan heat low over central Mali. The position of the ITD in Figure 3d highlights the northward flow of low-level moist air from the tropics into southern Algeria and northern Niger. Convective triggers in the form of the Hoggar and Aïr Mountain ranges are also present, and the rainfall from the early stages of the MCS can be seen in the TRMM rainfall estimate in Figure 3d.

### 3.2 Mesoscale Processes Important for MCS and Dust Plume Production

#### 3.2.1 Early Convective Events

**Figure 4:**
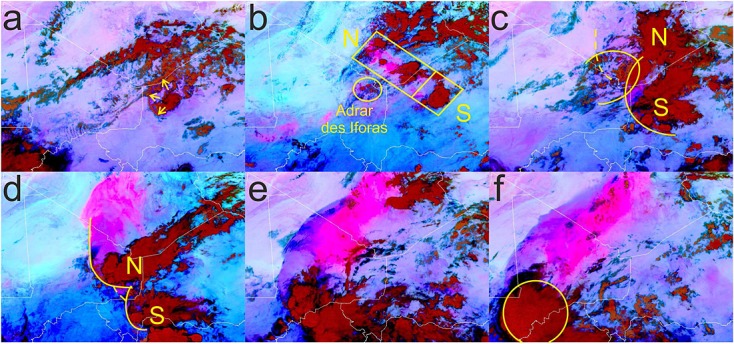
SEVIRI RGB dust images at (a) 0000 UTC 8 June, (b) 1500 UTC 8 June, (c) 0000 UTC 9 June, (d) 1200 UTC 9 June, (e) 0000 UTC 10 June, and (f) 0600 UTC 10 June 2010 over the region modeled using WRF. Marked are the cold pool flow from the early convective events in Figure 4a, three key regions of convective initiation during early development of the main MCS in Figure 4b, cold pool fronts with older cold pool edges being dashed in Figures 4c and 4d, and the circular cloud shield formed when the MCS develops into a mesoscale convective complex (MCC) type system in Figure 4f. Note that the northern part of the dust plume shown in Figure 4d is associated with the aged cold pool marked in Figure 4c.

On 7 June a number of smaller convective systems were triggered and collapsed 1 day prior to the initiation of the haboob-producing system. These grew to sizes that produced strong downdrafts and cold pools during the night of 7 and early morning of 8 June (Figure 4a). Rope clouds forming along the leading edges indicate that these spread north and west, crossing the Niger-Algeria and Niger-Mali borders (Figure 4a). This, combined with the northward bulge in the ITD (Figure 3d), is likely to have further advected moist air toward the Hoggar and Aïr Mountain ranges, encouraging convective activity on 8 June.

#### 3.2.2 Initiation and Development of MCS

On 8 June the first convective cells are initiated at approximately 1200 UTC in the vicinity of the Aïr Mountains and over the Algeria-Niger border. By 1500 UTC more cells start to develop over the southwestern edge of the Hoggar Mountains in what remains of the tropical plume cloud streak. This creates a line of convection with a northwest-southeast orientation (Figure 4b). Simultaneously, a number of smaller cells initiate southwest of the main axis of systems over the Adrar des Iforas, on the Malian side of the Mali-Algeria border (Figure 4b). The northern cells (N in Figure 4b) appear to move northwestward with little or no cloud formation along the western edge of the cold pool (marked with a dashed line in Figure 4c). The southern system (S in Figure 4b) produces a more well developed anvil and has a strong rope cloud along its cold pool leading edge. New convective cells are triggered intermittently along the southern edge of the cold pool from S. Convective triggering along the cold pool edge of N appears to require a stronger forcing. At approximately 2200 UTC the collision of cold pools from the Adrar des Iforas cells and S is responsible for the generation of a group of new convective cells in the northern part of the system. Later at 0000 UTC 9 June we can see a restrengthening of deep convective clouds on the SW side of N (Figure 4c). These develop rapidly, strengthening cold pool production from N, which flows west and north into the desert producing the southern half of the dust plume shown in Figure 4d, the northern part of which is associated with the aged cold pool marked with a dashed line in Figure 4c.

The initiation of new cells in different parts of the system at different times produces distinct lobes along the leading edge of the cold pool (Figure 4d). The cold air spreading away from more than one convective center along a single gust front produces conditions similar to the collision of two separate density currents. It is in these regions where new cells are preferentially generated. Other factors such as topographical lifting and localized convergence may also play a role. The need for a combination of uplift mechanisms before convective initiation is achieved and is likely a result of limited boundary layer moisture (not shown).

By 0000 UTC 10 June (Figure 4e) N and S have merged and later at 0600 UTC (Figure 4f) a well-defined round cloud shield has formed, suggesting the system's development into a mesoscale convective complex (MCC). Radiosonde launches show that there is a strong change in the direction of deep layer shear (0–8 km) between the stations in the north and south (see Figure 2 for station positions). The deep layer shear for 1200 UTC on 8 and 9 June at (1) In Salah is 19–23 m s^−1^in westerly and northwesterly directions, (2) Tamanrasset is 19–26 m s^−1^in a predominantly westerly direction, (3) Agadez is 8–10 m s^−1^ in an easterly direction, (4) Niamey 8–15 m s^−1^in predominantly easterly direction, and (5) Ouagadougou (only 1200 UTC 9 June available) 12.8 m s^−1^ in a northeasterly direction. It has been shown that deep layer shear has a strong influence on MCS organization, speed, and intensity [*Coniglio et al.*, 2006; *Cohen et al.*, 2007]. This suggests that after the system moves into the influence of the AEJ and deep layer shear is increased easterly, the MCS develops into a more organized MCC (Figures 4d–4f). An animated series of NASCube images (see Movie S1 in the supporting information) illustrates the mesoscale processes discussed in sections 3.2.1 and 3.2.2.

### 3.3 Synoptic-Scale Flow Responsible for Dust Transport

**Figure 5:**
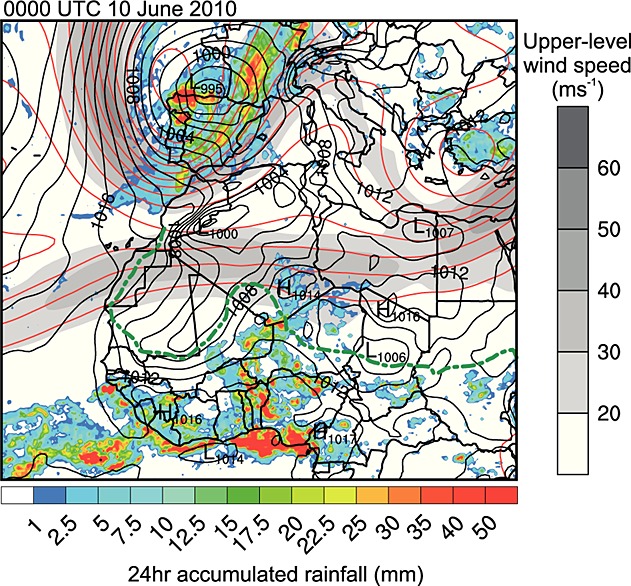
Same as Figure 3 but at 0000 UTC 10 June and including fields over southern Europe.

Figure 5 not only shows the same synoptic fields from ERA Interim and TRMM rainfall estimates as in Figure 3 but also shows southern Europe for 0000 UTC 10 June. The 200 hPa winds, 200 hPa geopotential, mean sea-level pressure, and TRMM rainfall show the presence of a midlatitude cyclone and trough over the Iberian Peninsula. The stream of the subtropical jet over West Africa is predominantly zonal, the northward bulge in the ITD is still present, and the rainfall associated with the MCS can be seen over Algeria, Niger, Mali, and Burkina Faso. The midlatitude cyclone produces a secondary low south of the Atlas Mountains through lee cyclogenesis. This deforms the Saharan heat low, which creates southwesterly flow over Algeria and northern Mali, which provides a mechanism for dust transport out of the Sahara toward the Mediterranean. SEVIRI dust images (not shown) show the stretching of the dust plume and northward transport of dust throughout 10 and 11 June.

## 4 Model Analysis

### 4.1 Effect of Early Convective Events on Boundary Layer Moisture

**Figure 6:**
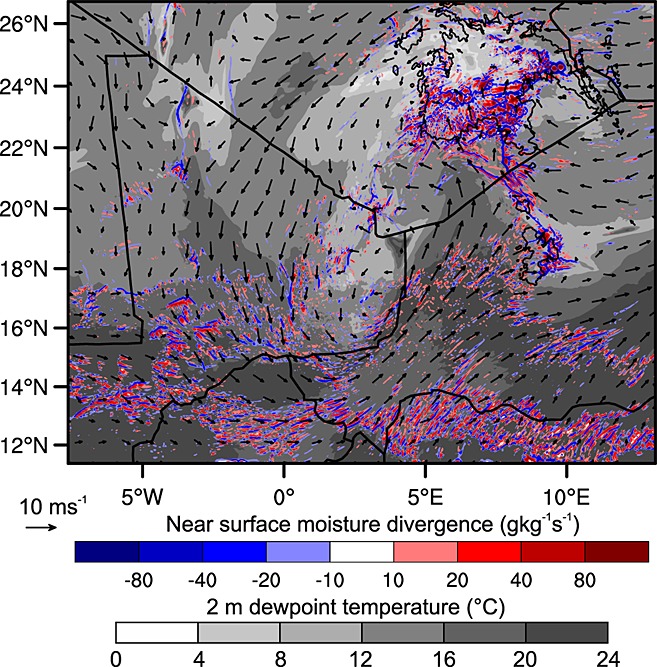
Moisture divergence calculated using 10 m winds and 2 m specific humidity (red and blue), 2 m dewpoint temperature (gray shading), and 10 m winds (vectors) from WRF simulation at 1200 UTC 7 June 2010 (approximate time of initiation of convective cells over the Hoggar Mountains). Note the circulations associated with the Libyan high and the heat low positioned over the Algeria/Mali/Niger border triple point, these produce the regions of moisture convergence and the tongue of moist air advected toward the Hoggar Mountains west of the Aïr Mountains.

As mentioned in section 3.2.1 convective cells initiated over the Aïr Mountains on the afternoon of 7 June created convective cold pools thought to favor convective triggering on 8 June. In the WRF simulation there is only very weak convective activity in the region of the Aïr at this time. However, there is widespread initiation over the Hoggar Mountain range. This is likely due to the orographic forcing of the mountains combined with a region of high moisture convergence associated with the Mediterranean cold surge and the restrengthening of the Saharan heat low (Figure 6). In the simulation this region of moisture convergence is strongest near the Hoggar Mountains, perhaps explaining the lack of convective development over the Aïr Mountains. These convective storms over the Hoggar Mountains produce a cold pool that spreads as far south as the northern Aïr Mountains. The simulated 1200 UTC boundary layer specific humidity over the grid point closest to Tamanrasset increases by 2.0 g kg^−1^ (5.1 g kg^−1^ to 7.1 g kg^−1^) between 7 and 8 June. Over the same period there was an increase of only 0.3 g kg^−1^ (12.8 g kg^−1^ to 13.1 g kg^−1^) over the grid point closest to Agadez in the southern Aïr Mountains. Similarly, 1200 UTC radiosonde launches from Tamanrasset show that the water vapor mixing ratio of the well-mixed boundary layer increase from 4.9 g kg^−1^ on 7 June to 6.4 g kg^−1^ on 8 June, an increase of 1.5 g kg^−1^over more than 3 km of the lower atmosphere (not shown). The 0900 UTC and 1200 UTC radiosonde launches from Agadez on 7 and 8 June, respectively, show little change to boundary layer moisture (about 12 g kg^−1^). This and the dewpoint temperatures at the two sites (Tamanrasset < 14°C and Agadez > 14°C) suggest that convection on 7 June is an important factor for boundary layer moistening north of the ITD, while in the monsoonal flow it had little impact.

### 4.2 Convective Initiation and Organization

**Figure 7:**
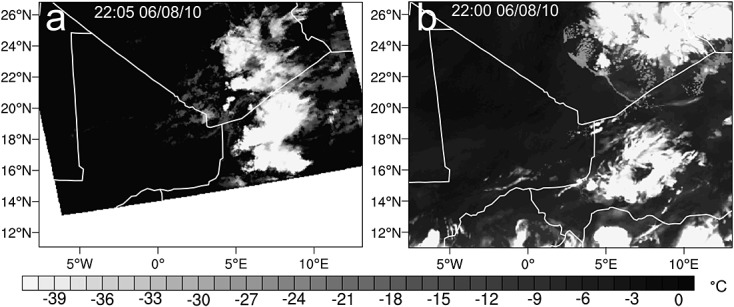
Comparison of position of deep cloud from MODIS and WRF simulation using 8.4–8.7 μm brightness temperature from MODIS and an estimated cloud top temperature found by using the WRF outgoing long-wave radiation and the Stefan Boltzman law.

**Figure 8:**
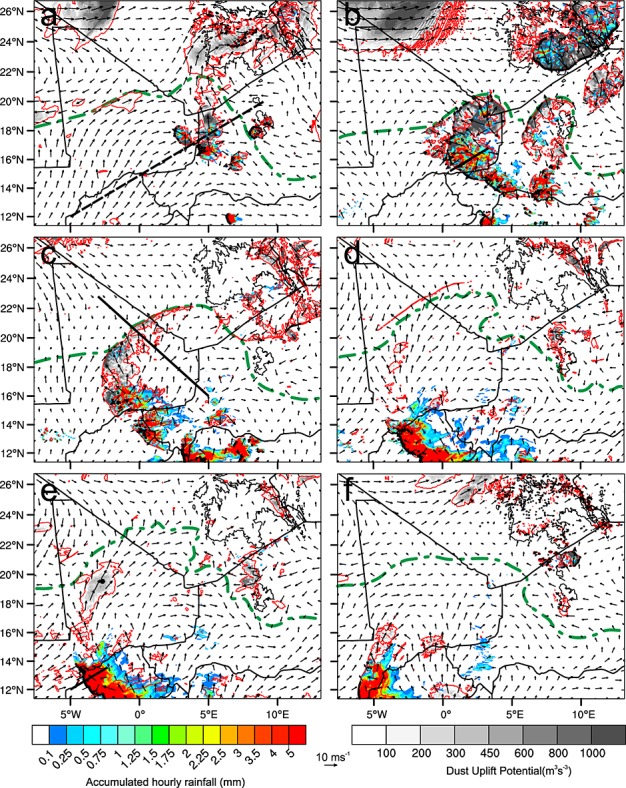
Temporal evolution of MCS and cold pool on 9–10 June 2010 as simulated by WRF. Shown is the 1 h accumulated rainfall (color shading), 10 m wind vectors, 7 m s^−1^ 10 m isotach (red contour), dust uplift potential described in section 4.3 (gray shading), 700 hPa vertical velocities exceeding 3 m s^−1^ (black dots), and ITD position ascertained from boundary layer dewpoint temperature (dashed green contour) at (a) 1200 UTC 9 June, (b) 1800 UTC 9 June, (c) 0000 UTC 10 June, (d) 0600 UTC 10 June, (e) 0800 UTC 10 June, and (f) 1200 UTC 10 June 2010. The thick black dashed line shown in Figure 8a is the common track along which the simulated MCS and the satellite observations were compared (Figure 9). The thick black lines in Figures 8b and 8e represent cross sections shown in Figure 10, and the line in Figure 8c represents the cross section used in Figure 1. The dot marked in Figure 8e represents the point chosen to represent nocturnal LLJ formation (Figure 3).

The triggering of convective cells over the Hoggar/Aïr region on 8 June occurs in the WRF simulation at approximately 1200 UTC. This agrees well with the initiation time seen in SEVIRI imagery (Figure 4b). Figure 7 shows the 2205 UTC 8.4–8.7 μm BT from MODIS and the 2200 UTC on 8 June estimated BT using WRF outgoing long-wave radiation and an emissivity of 1. The regions where convection has been produced in the simulation spatially differ to those seen in satellite imagery. There are two distinct regions of cold cloud tops (Figure 7b) rather than a number of cells oriented along a northwest-southeast axis (Figure 7a). In the simulation, active regions are over and west of the Aïr Mountains and over the Hoggar Mountains. The simulated system does not become organized as observed in satellite imagery. Instead, both groups of convective cells decay by 0000 UTC 9 June. This deviation from the observed system is not investigated here, but it is likely that the spatial differences between the simulated and observed convective initiation regions play a role in the lack of convective organization. The convective activity in the simulation is then reinitiated at 0600 UTC in the Niger-Mali-Algeria border region by the arrival of the cold pool (visible approximately 200 km northeast of the reinitiation region and spreading away from the Hoggar system in Figure 7b). This creates an approximate 6 h lag between the observed and simulated systems. Once triggered, this system rapidly becomes organized along a northwest-southeast axis and moves southwestward (the organized but still relatively small system can be seen in Figure 8a). The behavior displayed after the reinitiation (section 4.3) is consistent with that observed in satellite imagery (Figures 4 and 8) despite the temporal delay between the simulated and observed systems.

### 4.3 Behavior of Mature MCS

Figure 8 shows the development of the simulated MCS (Movie S2). Shown are vectors representing 10 m wind speed and direction, hourly accumulated rainfall, 7 m s^−1^ isotach at 10 m, updrafts exceeding 3 m s^−1^at 700 hPa, the position of the ITD derived from boundary layer dewpoint temperature, and the dust uplift potential (DUP) as described in *Marsham et al.* [2011]. The DUP is based on the parameterization of dust uplift by *Marticorena and Bergametti* [1995].

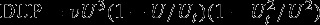
1where *ν* is bare soil fraction, *U*_*t*_ is a threshold wind speed (7 m s^−1^used in this study based on the values in *Chomette et al.* [1999] and *Marticorena et al.* [1997]), and *U* is the WRF 10 m winds. Its cubic nature means that it is highly sensitive to high winds. DUP is an idealized metric which isolates the role of meteorology in dust uplift from the land surface. *Cakmur et al.* [2004] observed that almost all areas of bare soil in this region lead to some dust uplift, and *Heinold et al.* [2013] showed that the reduction in uplift due to washout and soil moisture was less than 20% at most. Therefore, DUP is used here to identify the meteorological processes capable of lifting dust rather than providing a quantitative measure.

**Figure 9:**
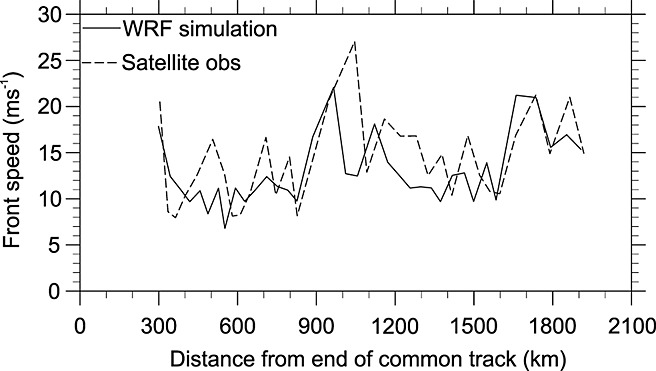
Comparison of front speed of simulated and observed MCS plotted against the distance from the northeast end of the common track (black dashed line in Figure 8a).

By 1200 UTC 9 June (Figure 8a) the convection has been retriggered by the cold pool mentioned in section 4.2 and has become organized producing new cells on its southwestern edge. A line of systems can be seen on a southeast-northwest orientation across the Mali-Niger border. At this time the cells are still relatively small and have not yet merged into a single organized system. They are positioned within the monsoon flow shown by the direction of the 10 m winds and the position of the ITD. Other convective cells appear to have been triggered over the Hoggar and Aïr Mountains at this time. Rainfall rates are high (exceeding 5 mm h^−1^) and high DUP values are produced within the radially spreading cold pools.

Six hours later at 1800 UTC 9 June (Figure 8b) the individual groups of cells have merged into a single organized system and continue to travel southwestward. The cold pools spread away from the initial downdraft regions and still display high DUP values. There is more rainfall production in the southern half of the system, and updraft regions (black dots) are mostly along the leading edge of the cold pool. However, some updrafts and the regions of highest rainfall are set back from the leading edge. The cold pools from the studied MCS and the system over the Aïr Mountains spread into the desert and push the ITD north. A region of high DUP is also present in the northwestern corner of the domain. This is thought to be linked to strong pressure gradients from the lee cyclone south of the Atlas Mountains. Such high DUP values would suggest significant dust uplift. SEVIRI RGB dust images (not shown) show some dust is raised by these winds; however, it is not widespread and has weak signal. This might be linked to differences between simulated and actual winds or low-level stability or atmospheric moisture preventing dust from being easily detected.

By 0000 UTC 10 June (Figure 8c) the cold pools have forced the position of the ITD as far north as 22°N and the MCS has continued to travel southwestward. The DUP within the cold pool has weakened, and regions above the 7 m s^−1^threshold are positioned close to the leading edge of the cold pool and near precipitating parts of the system. The cold pool flow has started to change with winds becoming less radial and beginning to turn to the right. Rainfall is focused in small intense regions well behind the leading edge of the cold pool edge and updrafts in excess of 3 m s^−1^at 700 hPa are similarly positioned.

At 0600 UTC 10 June (Figure 8d) the MCS has traveled farther southwest, rainfall along its northern edge has stopped, and rainfall in the southern half of the system becomes much stronger. Strong updrafts and heavy rainfall now occur along the entire leading edge of the system, and heavy rainfall is no longer centered in small intense regions suggesting a change in convective behavior. A region of weaker stratiform rain has also developed behind the main heavily precipitating region. By this stage the cold pool has aged and is not forcing moist air further into the desert. The flow within the aged cold pool is weaker with very few regions above 7 m s^−1^ and has become almost parallel to the edge of the cold pool. A region of interest is the narrow band of winds exceeding the dust uplift threshold beyond the edge of the aged cold pool. This feature is over 600 km long but less than 20 km wide and had DUP values in excess of 600 m^3^*s*^−3^. It is discussed further in section 5.3.

In Figures 8e and 8f (0800 and 1200 UTC 10 June, respectively) the MCS continues to travel southwestward, and rainfall rates, rainfall distribution, and updrafts are similar to Figure 8d. The wind direction within the aged cold pool continues to veer, and by 1200 UTC mixing due to solar heating of the surface has begun to reduce the dewpoint temperature perturbation and force the ITD position south. At 0800 UTC there is a large region of moderate DUP values inside the aged cold pool; the processes responsible for this will be discussed in section 5.2.

Also shown is a common track between the simulated and observed system (dashed line in Figure 8a). This is used to analyze the behavior of the MCS in both the simulation and in satellite imagery. The leading edge of the system is found by manually identifying the boundary of lifted dust and rope clouds in satellite imagery, and strong updrafts and equivalent and virtual potential temperature (*θ*_*e*_ and *θ*_*v*_, respectively) gradients in the WRF simulation. These variables were chosen as *θ*_*e*_is an adiabatically conserved quantity often used for air mass identification, while *θ*_*v*_ is a proxy for atmospheric density and is useful for identifying density driven flows. Figure 9 shows the front speed of the leading edge plotted against the distance away from the northeastern end of the track. This plotting method compensates for the temporal differences between the observed and simulated systems and accounts for topographic influences on convective initiation.

The velocities of the real and simulated fronts closely resemble each other. However, the first increase in front speed (at 800 km) is produced by different processes in the observed and simulated systems. From satellite imagery it is identified as the result of the rapid generation of convective cells to the north of the track. The cold pool surge associated with this development moves south, rapidly shifting the position of the cold pool boundary along the track. In the WRF simulation the leading edge is accelerated by the development of a number of cells ahead of the main system producing a region of uplift and cold pool air that becomes indistinguishable from the original front.

**Figure 10:**
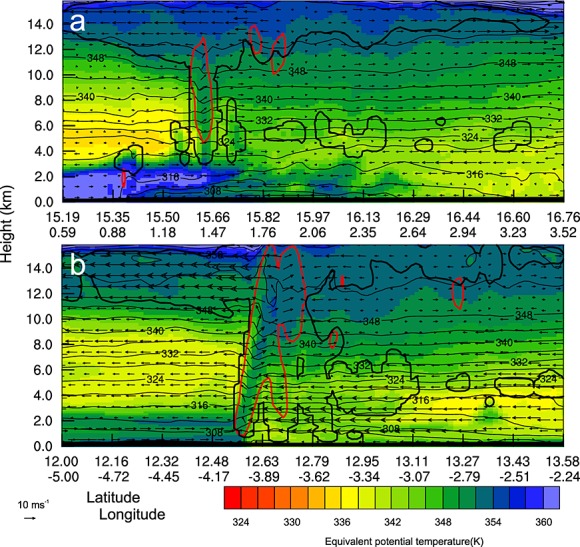
Cross sections through leading edge of MCS showing equivalent potential temperature (color shading), virtual potential temperature (thin black contours), outline of cloud including liquid and ice (thick black contour), and updrafts exceeding 2 m s^−1^ (thick red contours) at (a) 1800 UTC 9 June and (b) 0800 UTC 10 June 2010. The positions of the cross sections are shown in Figures 8b and 8e.

In both satellite imagery and the simulation the second increase in front velocity is accompanied by the development of a circular cloud shield (Figure 4f, WRF outgoing long-wave radiation for this time is not shown). As discussed earlier the MCS undergoes a change of convective mode with patterns of updrafts, rainfall intensity, and rainfall distribution changing as the system develops. Solid black lines in Figures 8b and 8e show the time and position of cross sections shown in Figure 10. These show wind, *θ*_*e*_, *θ*_*v*_, cloud, and updrafts exceeding 2 m s^−1^. Figure 10a shows that on 9 June the main cloud column is far behind the gust front (∼30 km), convective updrafts are weak, and the cloud base is at approximately 2.5 km above the surface. In contrast on 10 June (Figure 10b) the system fits the conceptual model of a MCS proposed by *Smull and Houze* [1987]. There is a region of strong updrafts produced along the gust front, evidence of a system relative rear inflow jet (not shown), and the cloud base is below 1 km. In the simulation there is only a small change in the low-level moisture ahead of the storm between these times (approximately 1 g kg^−1^) and a reduction in the convective available potential energy (approximately 2500 J kg^−1^ to 1500 J kg^−1^). The convective inhibition also remains low (below 150 J kg^−1^) suggesting the change in behavior is controlled by other factors.

One candidate for the change in MCS behavior is the shear profile. Shallow shear (over the depth of the cold pool) has been shown to have a strong influence on the behavior of the cold pool leading edge. However, in this case the shallow layer shear (0–2 km) is almost parallel to the gust front at the leading edge of the system and does not change much between 1800 UTC 9 June and 0800 UTC 10 June. As described in section 3.2.2, MCS behavior is also strongly linked to the deep layer shear. The deep vertical wind shear (0–8 km) ahead of the simulated system changes from values of 4–8 m s^−1^ for the earlier convective regime to over 20 m s^−1^for the regime with greater organization. The simulated pattern of both shear strength and direction agrees well with that seen from radiosonde data. Deep shear is generally westerly at latitudes to the north of 20°N with shear direction changing to easterly south of 15°N and strengthening toward the southern edge of the WRF domain (approximately 12°N). As mentioned in section 3.2.2, the shear at latitudes between 12°N and 15°N is associated with the presence of the AEJ. Therefore, the increased deep vertical shear associated with the presence of the AEJ largely governs the behavior of this MCS with the increasing depth of the monsoon layer encouraging the development of convective cells. The agreement of the simulation and observations with respect to (1) the timing of convective initiation before the decay, (2) the direction and speed of propagation once an organized system is generated, (3) the influence of deep layer shear on the behavior of the MCS, (4) the production of a very large cold pool, and (5) the agreement between the DUP metric and the regions observed to emit dust suggests that WRF has successfully modeled the dynamical features important to the production of a very large haboob such as that observed (Figure 4).

### 4.4 Processes Responsible for Enhanced Northward Flow of the Cold Pool

The simplest model of a cold pool is that of an instantaneous release of a column of dense fluid. As the column collapses it produces a density current which spreads radially away from a central point [*Roberts and Knippertz*, 2012]. *Hallworth et al.* [2001, and references therein] focus on this type of axisymmetric density currents in rotating frames of reference. Their findings include that density-driven flows, even in relatively weakly rotating systems, behave very differently to flows in nonrotating frames of reference. One of these features is a maximum radius (Rossby radius of deformation) reached when rotational effects begin to dominate over the inertial forces.

As mentioned in section 4.3 the simulated cold pool flow veers from a radial flow until it is almost parallel to the edge of the cold pool. This behavior is also suggested in the observed dust plume by the formation of structures consistent with horizontal shear instability (Figure 1) [*Kawashima*, 2011]. This shows that the cold pool is being strongly influenced by rotational effects. The maximum radius (assuming the flow is solely driven by density differences) is estimated using equations from *Hallworth et al.* [2001] and found to be from 415 km to 535 km. The observed and simulated cold pools spread far in excess of these values (∼800 km) suggesting that other factors are influencing the behavior of the cold pool. The similarity between the WRF simulation and satellite imagery suggests that whatever dynamical processes are responsible for the movement of this cold pool into the Sahara, they are successfully represented by using WRF.

#### 4.4.1 Synoptic-Scale Pressure Gradient

The large-scale pressure gradient is a potential source of additional northward motion. At 0000 UTC 10 June the pressure field (Figure 5) is in broad agreement with that seen in the WRF simulation (not shown). The main difference between the WRF simulation and ERA Interim pressure fields at this time is the presence of a mesoscale high in WRF of approximately 2 hPa associated with the cold pool. The development of the lee cyclone discussed in section 3.3 southeast of the Atlas Mountains in Morocco produces an elongated heat low along a northeast-southwest axis. A mean sea-level pressure difference of 10 hPa is present between the leading edge of the cold pool and the low pressure center of the lee cyclone. This not only enhances the relative depth of the heat low but also displaces its center much farther north than usually seen. This creates a northwestward pressure gradient force over a much greater latitudinal extent of the Sahara. This additional forcing might help to explain the propagation of the cold pool deep into the desert.

#### 4.4.2 Shallow Convection on Saharan Edge of Cold Pool

**Figure 11:**
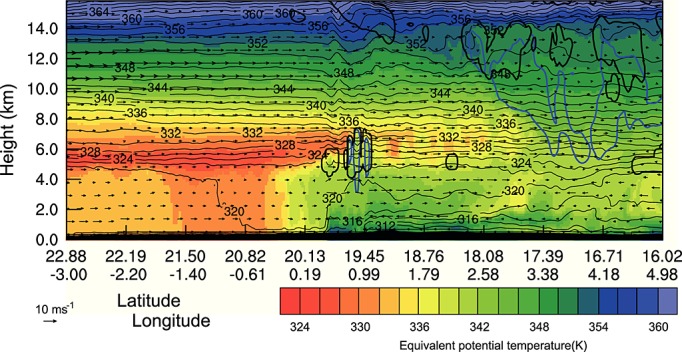
Cross section through cold pool traveling into the desert showing equivalent potential temperature (color shading), virtual potential temperature (thin black contours), outline of cloud including liquid and ice (thick black contour), and outline of precipitation (thick blue contours) at 2100 9 June. The position of the cross sections are shown in Figure 8.

**Figure 12:**
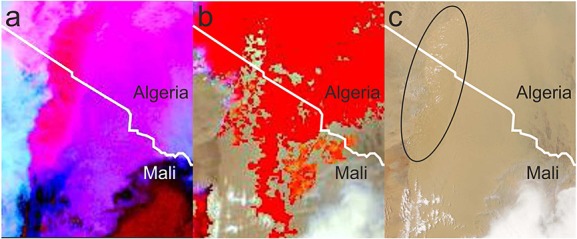
Comparison of (a) SEVIRI RGB pink dust, (b) NASCube visible and temperature anomaly (red denotes regions with a large temperature anomalies overlying color imagery), and (c) MODIS visual satellite products at 1330 UTC 9 June 2010 showing the difference in representation of shallow cumulus clouds formed over a region of raised dust (see section 2.1 for description of products). The area highlighted shows the position of small clouds visible in MODIS imagery.

Another factor that might explain the extent of the cold pool ingress into the desert is the generation of additional convective cells along the leading edge of the cold pool. When considering the movement of an MCS, the limitations on the distance traveled are not related to a theoretical maximum outflow distance of the initial cold pool. If new convective cells are triggered along a section of the leading edge traveling into the desert, the system is no longer similar to a single release density current as seen in laboratory experiments. Figure 11 is a cross section through the head of the cold pool positioned roughly perpendicular to its direction of travel (solid black line marked in Figure 8c). Contours of *θ*_*e*_ and *θ*_*v*_ show the position of different air masses, vectors show winds parallel to the cross section, and thick black and blue contours outline regions of cloud and precipitation, respectively. Shallow convective cells near the edge of the cold pool (between 3.5 and 7 km above the surface and 20 to 30 km across) produce precipitation that evaporates in the boundary layer. Cells producing precipitation which reaches the surface can also be seen close to the northern edge of the cold pool in Figure 8c. These have the potential to alter the expected behavior of a density current by refreshing the cooled air in the cold pool. Clouds similar to those shown in Figure 11 are likely to be overlooked in satellite imagery as they are shallow, and their tops do not significantly differ in temperature to the lofted dust below (Figure 12). The clouds shown in Figure 12 have a limited horizontal extent (approximately 3 km), this means that they are difficult to see even in 1 km pixel geostationary satellite imagery (Figures 12a and 12b). These clouds are evident in Figure 12c in the 250 m resolution MODIS true color imagery while they are difficult to see in either the NASCube thermal anomaly or SEVIRI dust imagery due to limitations associated with their dependence on BT and BT differences and the similar temperature of cloud and lofted dust. The grid spacing of the WRF simulation is too coarse to model the horizontal scale of clouds seen in Figure 12c; however, it is thought that the production of shallow, precipitation producing convective cells is a realistic process and could help to force cold pool air into the desert.

## 5 Dust Uplift Processes

By identifying meteorological mechanisms which produce DUPs in the simulation, we aim to identify important dynamical processes for dust deflation. Three dust uplift processes are identified in the simulation and described in the following subsections: (1) high winds associated with the leading edge of the cold pool, (2) an enhanced nocturnal LLJ mixed to the surface in the morning, and (3) the production of an internal bore on the nocturnal boundary layer (NBL).

### 5.1 Turbulence Along Cold Pool Leading Edge

Despite the fact that observed and simulated cold pools cannot be considered simple axisymmetric single release density currents (section 4.4), here we assume that the mesoscale dynamics of the head of the observed cold pool behave similarly to two-dimensional laboratory experiments discussed in *Simpson*[1997]. Fluid overrun by advancing cold air rises through the flow and produces a turbulent head region. This turbulence creates intense gusts which can be sufficiently strong to deflate dust in arid regions, creating a haboob. As a dust uplift mechanism turbulence in the head of a convective cold pool is well documented [*Chen and Fryrear*, 2002] and is the most commonly attributed dust uplift mechanism ascribed to cold pools (see section 1). The grid spacing in the simulation is not sufficient to resolve the small-scale turbulent eddies; therefore, high winds in the model are produced by the mesoscale pressure gradient.

In Figures 8a and 8b large areas of strong surface winds are found behind the northward traveling edge of the simulated cold pool. Over time the region of high DUP gets narrower and more closely associated with the leading edge. This is caused by the weakening of winds within the cold pool as downdraft production ceases and the density current slows. By 0600 UTC (Figure 8d) only a very small section of the cold pool front still has wind speeds exceeding 7 m s^−1^suggesting that little dust uplift is produced by the leading edge of the cold pool at this time. DUP values are also likely to be reduced close to downdraft producing regions as the system travels south into vegetated regions.

### 5.2 Enhancement of Nocturnal LLJ

As mentioned in section 4.3 an area within the aged cold pool produces winds above the dust uplift threshold from 0700 to 1100 UTC 10 June (Figure 8e). This is linked to the production of a nocturnal LLJ. Momentum from this LLJ is then mixed to the surface by the growth of the boundary layer the following morning. As mentioned in section 1 LLJs are an important dust uplift mechanism in the Sahara. They have not usually been associated with cold pools; however, recent work by *Heinold et al.* [2013] and *Marsham et al.* [2013a] has highlighted a connection between the two. The effect seen in the WRF simulation is very similar to that identified in high-resolution Met Office Unified Model runs by *Heinold et al.* [2013]. DUP values over a large area within the aged cold pool in Figure 8e are 300–600 m^3^s^−3^, suggesting a link between the presence of the cold pool and an enhancement of the processes forming the nocturnal LLJ.

**Figure 13:**
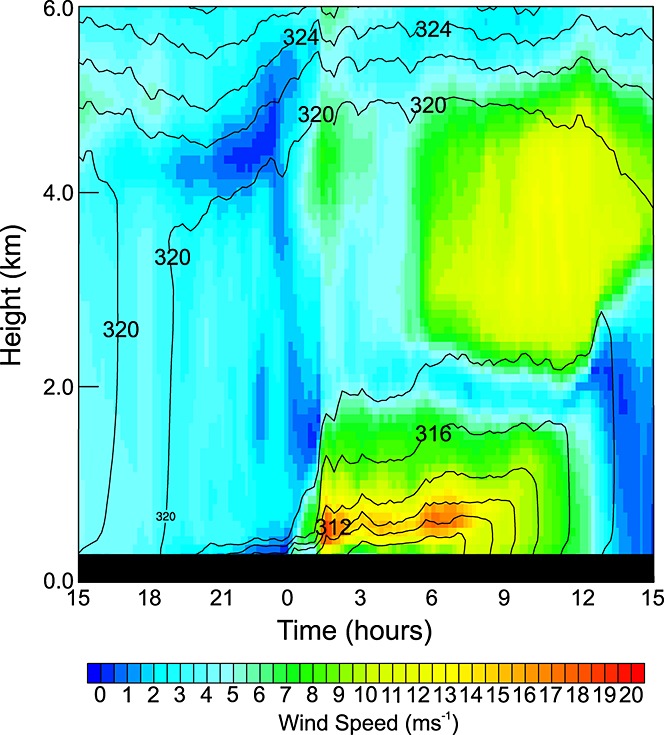
Time series of wind speed and stability in the boundary layer from 1500 UTC 9 June to 1500 UTC 10 June 2010. Wind speed is represented by the color shading and stability is represented through contours of virtual potential temperature (black contours).

Figure 13 shows wind speed and *θ*_*v*_for the boundary layer during the cold pool passage for the point marked in Figure 8e. The profile shows the increasing depth of the stable layer prior to the arrival of the cold pool just before 0100 UTC and the production of a nocturnal LLJ reaching 8 m s^−1^. The passage of the leading edge of the cold pool at 0100 UTC produces strong winds, increased *θ*_*v*_, and higher levels of water vapor (not shown) in the lowest 1.5 km of the atmosphere. After the leading edge of the flow has passed, the strongest winds are between 200 m and 600 m above the surface, reaching 17 m s^−1^. Between 0100 and 0500 UTC the wind speeds at this elevation reduce slightly to between 14 m s^−1^ and 16 m s^−1^, most likely associated with weaker flow to the rear of the leading edge. The highest wind speeds are found at the interface between the cold pool and the residual mixed/monsoon air above. This marks the most stable layer of the lower atmosphere. The near surface winds are still relatively strong (>5 m s^−1^) but not as high as the 7 m s^−1^threshold velocity chosen for dust uplift suggesting that the air is not stable enough for a complete decoupling. From 0500 to 0700 UTC there is a further acceleration of the jet to as high as 18 m s^−1^. This may at least be partly due to an inertial oscillation [*Fiedler et al.*, 2013], where supergeostrophic winds can be achieved due to the effects of coriolis acceleration. Also, at this time the synoptic-scale pressure gradient force and the cold pool flow are perpendicular, suggesting that the superposition of cold pool momentum and the background flow is a factor in the production of this strong nocturnal LLJ. At 0700 UTC the drop in near-surface values of *θ*_*v*_indicate that the low-level inversion is being eroded by surface heating. Higher winds at the surface at this time indicate that nocturnal LLJ momentum has started to be mixed throughout the growing boundary layer. This process continues throughout the morning producing surface wind speeds in excess of 7 m s^−1^ for over 4 h.

### 5.3 Interaction Between Cold Pool and Developing NBL

The narrow region of high DUP values ahead of the aged cold pool mentioned in section 4.3 (Figure 8d) is produced by the formation of an internal bore on the NBL. A cold pool moving through the growing NBL is considered here to be dynamically similar to a density current flowing in a stratified environment. *Simpson* [1997] noted that if the depth of a density current is less than 4 times the depth of the stable layer, then the system can be described as being partially blocked and an internal bore will be generated. After sunset at approximately 1800 UTC 9 June the NBL begins to form and grows in depth. At approximately 2300 UTC 9 June the NBL reaches a depth approximately 1/4 the height of the density current. At this time the behavior of the cold pool head begins to change. While at 2300 UTC the cold pool still seems unimpeded by the presence of the NBL (see Movie S3 in the supporting information), the head of the flow becomes depressed and the NBL air is forced over the flow by 0000 UTC June, similar to the smooth hump that envelops a density current head described in *Simpson* [1997]. An internal bore formed by the cold pool then propagates ahead of the edge of the flow at approximately 0500 UTC. During this time the cold pool flow changes behavior significantly including the development of what *Hallworth et al.* [2001] describe as a nose-down profile similar to that seen in laboratory density currents in rotating frames of reference. There is also a rapid deceleration of the cold pool front between 0500 and 0700 UTC which is consistent with a density-driven flow reaching its Rossby radius of deformation. The deceleration of the cold pool allows the bore produced at its front to propagate into the desert.

The type of bore formed is known to be controlled by the ratio of bore height to stable layer height (*h*_0_/ *h*_1_) [*Simpson*, 1997]. A value between 1 and 2 produces a smooth undular bore, a value between 2 and 4 produces a bore with some turbulent mixing, and a value higher than 4 is dynamically very similar to a gravity current. Before solar heating of the surface begins to erode the NBL, the amplitude of the simulated bore is large compared to the depth of the NBL. Therefore, it is likely that a significant amount of turbulence would be created at the rear of such a bore. It is proposed that a turbulent bore has the potential to raise dust from the surface as the simulated DUP along much of the bore is in excess of 600 m^3^s^−3^ (not shown). This mechanism appears to be similar to a case in southwest Algeria (17 and 18 June 2011) as part of the Fennec field campaign [*Hobby et al.*, 2013], where winds associated with the bore were in excess of 7 m s^−1^ and dust was observed in the SEVIRI RGB dust product.

## 6 Summary and Conclusions

To the best of our knowledge this study is the first to successfully simulate a case of a very large convectively generated cold pool in the central Sahara (nearing 1000 km long). This study simulates the dynamical conditions of a MCS triggered over the Aïr and Hoggar Mountain ranges on 8 June 2010 and the cold pool it creates. Including analysis of how these conditions are likely to impact Saharan dust emission. Despite temporal discrepancies in the development of the MCS between our simulation and observations, it is argued that the two are sufficiently similar such that useful conclusions can be drawn about this case and similar systems.

**Figure 14:**
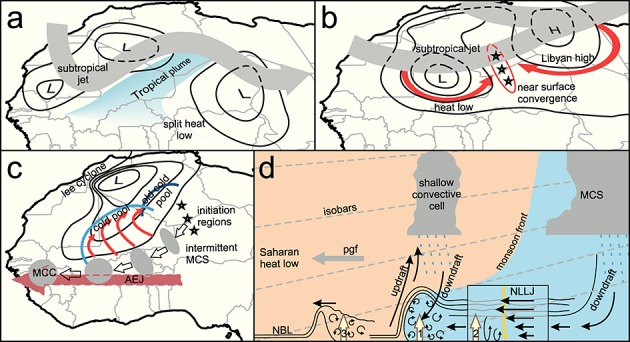
Schematic detailing both the synoptic-scale and mesoscale processes that led to the production of the large MCS and dust plume on 10 June 2010. (a) Formation of a tropical plume and the effect on the Saharan heat low (section 3.1). (b) Restrengthening of the heat low and generation of a high over Libya creating low-level convergence (section 3.1). (c) Initiation and development of the MCS, its production of cold pools, and the influence of the lee cyclone and the AEJ (sections 3.2.2, 4.2, and 4.3). (d) The mesoscale features that lead to dust uplift. Shown in Figures 14a–14c are mean sea-level pressure features (black isobars), subtropical jet (large grey arrows), tropical plume (blue shading), low-level winds (red arrows), cold pool edges (blue lines), convective initiation regions (black stars), the African easterly jet (large light red arrow), and the position of the MCS (grey ellipses). Shown in Figure 14d is the production of a convective cold pool spreading into the desert including the pressure gradient force (pgf) produced by the lee cyclone, shallow convective cells, the monsoon front, and the three dust uplift processes identified. These are (1) strong winds at the leading edge of the cold pool flow, (2) nocturnal LLJ within the aged cold pool, and (3) formation of an internal bore on the NBL. See section 6 for a more detailed discussion.

Figure 14 is a schematic showing the main processes responsible for the production of the dust plume in this case study. The synoptic-scale environment plays an important role in creating the conditions for the production of the MCS. An upper level trough and wave on the subtropical jet are responsible for the production of conditions dynamically similar to a tropical plume (Figure 14a). The persistent and precipitating clouds created likely lead to the disruption of the heat low, splitting it in two, as well as moistening the desert air. Subsequent straightening of the subtropical jet and the development of an upper level trough over the eastern Mediterranean are linked to the development of a high over Libya (Figure 14b). In the simulation, flows associated with the restrengthening heat low and Libyan high produce a convergence zone over the Hoggar and Aïr Mountains. Convergence, large-scale moisture advection, and high terrain create favorable conditions for convective triggering.

On 7 June smaller convective systems are triggered, producing cold pools that spread over the Hoggar/Aïr regions and extend the area of moist southerlies. These cold pools increase levels of moisture in the boundary layer and encourage the production of further convective cells. At 1200 UTC on 8 June the first cells of the MCS are triggered (Figure 14c). Cells produced farther south along the northwest-southeast axis of the system develop more rapidly and produce more new cells than those in the north. The marginal nature of new cell triggering early in the system's development is likely due to low boundary layer moisture. The initiation of new cells at this stage seems reliant on a number of factors, making the generation of new cells more intermittent. The system's behavior changes radically when it moves into a region of strong easterly deep layer shear and increasing boundary layer moisture. The shear (provided by the AEJ (Figure 14c)) produces a convective system which closely resembles a MCC with well-organized updrafts, continuous convective initiation along the cold pool edge, a large area of stratiform rain behind the region of heavy precipitation, and evidence of a descending rear inflow jet.

Comparison between the simulated and observed cold pools and theoretical density currents has shown that the analogy of a simple single release density current is not appropriate. Factors which are likely to make this case behave differently to the simple model are (1) the southwestward movement of the MCS while it continues to generate convective downdrafts, (2) the presence of a strong northward pressure gradient over almost the entire latitudinal extent of the Sahara, generated by lee cyclogenesis south of the Atlas Mountains, and (3) shallow, precipitating, convective cells along the northern edge of the cold pool, repeatedly refreshing the evaporatively cooled air in the flow.

Later, the dust plume is deformed by the background flow produced by the deformation of the Saharan heat low by lee cyclogenesis. This deformation is present from 9 to 11 June, and the large-scale circulation induced is able to transport dust over long distances.

To identify dust uplift processes, a measure of the meteorological impact on dust uplift (DUP) was used, as it separates the meteorological influences of dust deflation from the land surface. Three main processes were identified from the WRF simulation (Figure 14d):

1. High winds at the leading edge of the cold pool.
2. The mixing of high momentum air to the surface from an enhanced nocturnal LLJ within the aged cold pool once solar heating begins to erode the nocturnal stable layer. This process is not well represented in literature but is dynamically similar to that proposed in *Heinold et al.* [2013].
3. The generation of an internal bore on the NBL, by the cold pool, which then propagates into the desert with the stable layer acting as a wave guide. As the amplitude of the bore is large compared to the depth of the NBL, the bore is thought to be turbulent and in the simulation produces high values of DUP. This represents a previously unrecognized mechanism for dust uplift. Much lower total DUP values than the cold pool are generated but this mechanism extends the distance away from a MCS that dust uplift can potentially occur.

The results presented here address the specific mechanisms that produced this unusual dust plume. Further work on this type of system is needed to identify if similar synoptic-scale conditions are present for other convective, dusty episodes. The mesoscale processes mentioned should also be investigated to ascertain the influence that the suggested mechanisms have on cold pool behavior. This will help show whether the findings in this study are applicable to a greater number of large dust deflation events. Other areas that should be investigated are the relative importance of the different dust uplift processes identified, including the amount of dust raised by particular phenomena and how commonly they occur. Also, the differences between different reanalyses and operational analyses should be investigated in this region of the world due to the large dependence on data used to initialize the simulations discussed in section 2.3 compared to changes made to model physics.

## Supporting Information

Read Me

## References

[b1] Berry G, Thorncroft C D (2005). Case study of an intense African easterly wave. Mon. Weather Rev.

[b2] Brazel A J, Nickling W G (1986). The relationship of weather types to dust storm generation in Arizona (1965–1980). J. Climatol.

[b3] Brindley H, Knippertz P, Ryder C, Ashpole I (2012). A critical evaluation of the ability of the Spinning Enhanced Visible and Infrared Imager (SEVIRI) thermal infrared red-green-blue rendering to identify dust events: Theoretical analysis. J. Geophys. Res.

[b4] Bristow C S, Hudson-Edwards K A, Chappell A (2010). Fertilizing the Amazon and equatorial Atlantic with West African dust. Geophys. Res. Lett.

[b5] Burton R R, Devine G M, Parker D J, Chazette P, Dixon N, Flamant C, Haywood J M (2013). The Harmattan over West Africa: Nocturnal structure and frontogenesis. Q. J. R. Meteorol. Soc.

[b6] Byers H R (1949). Structure and dynamics of the thunderstorm. Science.

[b7] Cakmur R V, Miller R L, Torres O (2004). Incorporating the effect of small scale circulations upon dust emission in an AGCM. J. Geophys. Res.

[b8] Chen W, Fryrear D W (2002). Sedimentary characteristics of a haboob dust storm. Atmos. Res.

[b9] Chomette O, Legrand M, Marticorena B (1999). Determination of the wind speed threshold for the emission of desert dust using satellite remote sensing in the thermal infrared. J. Geophys. Res.

[b10] Cohen A E, Coniglio M C, Corfidi S F, Corfidi S J (2007). Discrimination of mesoscale convective system environments using sounding observations. Weather Forecast.

[b11] Coniglio M C, Stensrud D J, Wicker L J (2006). Effects of upper-level shear on the structure and maintenance of strong quasi-linear mesoscale convective systems. J. Atmos. Sci.

[b12] Cuesta J, Lavaysse C, Flamant C, Mimouni M, Knippertz P (2010). Northward bursts of the West African monsoon leading to rainfall over the hoggar massif, Algeria. Q. J. R. Meteorol. Soc.

[b13] Dee DP (2011). The ERA-Interim reanalysis: Configuration and performance of the data assimilation system. Q. J. R. Meteorol. Soc.

[b14] Derbyshire E (2007). Natural minerogenic dust and human health. AMBIO J. Hum. Environ.

[b15] Emmel C, Knippertz P, Schulz O (2010). Climatology of convective density currents in the southern foothills of the Atlas Mountains. J. Geophys. Res.

[b16] Engelstaedter S, Washington R (2007). Atmospheric controls on the annual cycle of North African dust. J. Geophys. Res.

[b17] Farquharson M (1937). Haboobs and instability in the sudan. Q. J. R. Meteorol. Soc.

[b18] Fiedler S, Schepanski K, Heinold B, Knippertz P, Tegen I (2013). Climatology of nocturnal low-level jets over North Africa and implications for modeling mineral dust emission. J. Geophys. Res. Atmos.

[b19] Flamant C, Chaboureau J P, Parker D J, Taylor C M, Cammas J P, Bock O, Timouk F, Pelon J (2007). Airborne observations of the impact of a convective system on the planetary boundary layer thermodynamics and aerosol distribution in the inter-tropical discontinuity region of the West African monsoon. Q. J. R. Meteorol. Soc.

[b20] Freeman M (1952).

[b21] Fröhlich L, Knippertz P, Fink A H, Hohberger E (2013). An objective climatology of tropical plumes. J. Clim.

[b22] Garcia-Carreras L, Marsham J H, Parker D J, Bain C L, Milton S, Saci A, Salah-Ferroudj M, Ouchene B, Washington R (2013). The impact of convective cold pool outflows on model biases in the Sahara. Geophys. Res. Lett.

[b23] Goudie A S (2009). Dust storms: Recent developments. J. Environ. Manage.

[b24] Goudie A S, Middleton N J (2001). Saharan dust storms: Nature and consequences. Earth Sci. Rev.

[b25] Hallworth M A, Huppert H E, Ungarish M (2001). Axisymmetric gravity currents in a rotating system: Experimental and numerical investigations. J. Fluid Mech.

[b26] Hamilton R A, Archbold J W, Douglas C K M (1945). Meteorology of Nigeria and adjacent territory. Q. J. R. Meteorol. Soc.

[b27] Heinold B, Knippertz P, Marsham J H, Fiedler S, Dixon N S, Schepanski K, Laurent B, Tegen I (2013). The role of deep convection and nocturnal low-level jets for dust emission in summertime West Africa estimates from convection-permitting simulations. J. Geophys. Res. Atmos.

[b28] Hobby M (2013). The Fennec automatic weather station (AWS) network: Monitoring the Saharan climate system. J. Atmos. Oceanic Technol.

[b29] Huffman G J, Bolvin D T, Nelkin E J, Wolff D B, Adler R F, Gu G, Hong Y, Bowman K P, Stocker E F (2007). The TRMM multisatellite precipitation analysis (TMPA): Quasi-global, multiyear, combined-sensor precipitation estimates at fine scales. J. Hydrometeor.

[b30] Jickells TD (2005). Global iron connections between desert dust, ocean biogeochemistry, and climate. Science.

[b31] Kawashima M (2011). Numerical study of horizontal shear instability waves along narrow cold frontal rainbands. J. Atmos. Sci.

[b32] Knippertz P (2008). Dust emissions in the West African heat trough—The role of the diurnal cycle and of extratropical disturbances. Meteorol. Z.

[b33] Knippertz P, Martin J E (2005). Tropical plumes and extreme precipitation in subtropical and tropical West Africa. Q. J. R. Meteorol. Soc.

[b34] Knippertz P, Todd M C (2010). The central west Saharan dust hot spot and its relation to African easterly waves and extratropical disturbances. J. Geophys. Res.

[b35] Knippertz P, Todd M C (2012). Mineral dust aerosols over the Sahara: Meteorological controls on emission and transport and implications for modeling. Rev. Geophys.

[b36] Knippertz P, Deutscher C, Kandler K, Müller T, Schulz O, Schütz L (2007). Dust mobilization due to density currents in the Atlas region: Observations from the Saharan Mineral Dust Experiment 2006 field campaign. J. Geophys. Res.

[b37] Knippertz P, Trentmann J, Seifert A (2009). High resolution simulations of convective cold pools over the northwestern Sahara. J. Geophys. Res.

[b38] Lawson T (1971). Haboob structure in Khartoum. Weather.

[b39] Marsham J H, Parker D J, Grams C M, Taylor C M, Haywood J M (2008). Uplift of Saharan dust south of the intertropical discontinuity. J. Geophys. Res.

[b40] Marsham J H, Knippertz P, Dixon N S, Parker D J, Lister G M S (2011). The importance of the representation of deep convection for modeled dust-generating winds over West Africa during summer. Geophys. Res. Lett.

[b41] Marsham JH (2013a). Meteorology and dust in the central Sahara: Observations from Fennec supersite-1 during the June 2011 Intensive Observation Period. J. Geophys. Res. Atmos.

[b42] Marsham J H, Dixon N S, Garcia-Carreras L, Lister G M S, Parker D J, Knippertz P, Birch C E (2013b). The role of moist convection in the West African monsoon system: Insights from continental-scale convection-permitting simulations. Geophys. Res. Lett.

[b43] Marticorena B, Bergametti G (1995). Modeling the atmospheric dust cycle: 1. design of a soil-derived dust emission scheme. J. Geophys. Res.

[b44] Marticorena B, Bergametti G, Aumont B, Callot Y, N'Doumé C, Legrand M (1997). Modeling the atmospheric dust cycle: 2. Simulation of Saharan dust sources. J. Geophys. Res.

[b45] Membery D (1985). A gravity wave haboob?. Weather.

[b46] Miller S D, Kuciauskas A P, Liu M, Ji Q, Reid J S, Breed D W, Walker A L, Mandoos A A (2008). Haboob dust storms of the southern Arabian peninsula. J. Geophys. Res.

[b47] Mitsuta Y, Hayashi T, Takemi T, Hu Y, Wang J, Chen M (1995). Two severe local storms as observed in the arid area of northwest China. J. Meteorol. Soc. Jpn. Ser. II.

[b48] Ohde T, Siegel H (2010). Biological response to coastal upwelling and dust deposition in the area off Northwest Africa. Cont. Shelf Res.

[b49] Reinfried F, Tegen I, Heinold B, Hellmuth O, Schepanski K, Cubasch U, Huebener H, Knippertz P (2009). Simulations of convectively driven density currents in the Atlas region using a regional model: Impacts on dust emission and sensitivity to horizontal resolution and convection schemes. J. Geophys. Res.

[b50] Roberts A, Knippertz P (2012). Haboobs: Convectively generated dust storms in West Africa. Weather.

[b51] Schepanski K, Tegen I, Laurent B, Heinold B, Macke A (2007). A new Saharan dust source activation frequency map derived from MSG-SEVIRI IR-channels. Geophys. Res. Lett.

[b52] Shao Y, Wyrwoll K-H, Chappell A, Huang J, Lin Z, McTainsh G H, Mikami M, Tanaka T Y, Wang X, Yoon S (2011). Dust cycle: An emerging core theme in Earth system science. Aeolian Res.

[b53] Simpson J (1997). Gravity Currents: In the Environment and the Laboratory.

[b54] Skamarock W C, Klemp J B (2008). A time-split nonhydrostatic atmospheric model for weather research and forecasting applications. J. Comput. Phys.

[b55] Slingo A (2006). Observations of the impact of a major Saharan dust storm on the atmospheric radiation balance. Geophys. Res. Lett.

[b56] Smull B F, Houze R A (1987). Rear inflow in squall lines with trailing stratiform precipitation. Mon. Weather Rev.

[b57] Solomos S, Kallos G, Mavromatidis E, Kushta J (2012). Density currents as a desert dust mobilization mechanism. Atmos. Chem. Phys.

[b58] Sterk G (2003). Causes, consequences and control of wind erosion in Sahelian Africa: A review. Land Degrad. Dev.

[b59] Strong C L, Parsons K, McTainsh G H, Sheehan A (2011). Dust transporting wind systems in the lower Lake Eyre Basin, Australia: A preliminary study. Aeolian Res.

[b60] Sultan B, Labadi K, Gugan J-F, Janicot S (2005). Climate drives the meningitis epidemics onset in West Africa. PLoS Med.

[b61] Sutton L (1925). Haboobs. Q. J. R. Meteorol. Soc.

[b62] Takemi T (1999). Structure and evolution of a severe squall line over the arid region in northwest China. Mon. Weather Rev.

[b63] Takemi T (2005). Explicit simulations of convective-scale transport of mineral dust in severe convective weather. J. Meteorol. Soc. Jpn. Ser. II.

[b64] Thompson G, Field P R, Rasmussen R M, Hall W D (2008). Explicit forecasts of winter precipitation using an improved bulk microphysics scheme. part II: Implementation of a new snow parameterization. Mon. Weather Rev.

[b65] Vizy E K, Cook K H (2009). A mechanism for African monsoon breaks: Mediterranean cold air surges. J. Geophys. Res.

[b66] Wang W (2012). Advanced Research WRF (ARW) Version 3 Modeling System User's Guide.

[b67] Washington R, Todd M, Middleton N J, Goudie A S (2003). Dust-storm source areas determined by the total ozone monitoring spectrometer and surface observations. Ann. Assoc. Am. Geog.

