# Supplementary material for: The formation of a large summertime Saharan dust plume: Convective and synoptic-scale analysis
Source: J Geophys Res Atmos. 2014 Feb 26;119(4):1766–85. doi: 10.1002/2013JD020667 (PMC4379907; doi:10.1002/2013JD020667)
Supplement: Supplementary file 1 — Read Me [file jgrd0119-1766-sd1.doc]

Auxiliary material for

The Formation of a Large Summertime Saharan Dust Plume: Convective and Synoptic-scale Analysis

Alexander J. Roberts

Peter Knippertz

(School of Earth and Environment, University of Leeds, Leeds, UK)

Journal of Geophysical Research: Atmospheres, 2013

Introduction

This supporting material is provided to show the development of the meso-scale convective system (MCS) and the dust plume associated with it. All three figures described here are .gif animations allowing the reader to see the processes which are described in detail in the text. The data used to make ms01.gif are from the NASCube project (described in section 2.1 of the main text) the images shown are the true color/pseudo true color images with temperature anomaly overlaid and were accessed from <http://nascube.univ-lille1.fr/>. The data used to make ms02 and ms03 are from the WRF simulation. ms02 shows an animation of images as shown in Figure 8 in the main text from the start of the simulation to 96 hours after initiation. ms03 shows an animation of cross sections of wind, specific humidity, and virtual potential temperature from 0 to 2 km above sea level following the leading edge of the cold pool as it travels into the desert. The leading edge is always kept in the center of the frame and the cross sections all lie along the line shown in Figure 8c. ms03 shows the slowing of the cold pool front and the formation of a bore on the nocturnal boundary layer as discussed in section 5.3 of the main text.

1 ms01.gif The initiation and development of the MCS being studied and the resultant dust plume shown using NASCube combined true color/pseudo color and thermal anomaly (bright colors) images. The animation runs from 0000 UTC 07 June to 0000 UTC 11 June 2010 and shows the region over which the simulation was performed. Note the growth and collapse of smaller cells on the evening of 07 June and then the initiation and development of the larger MCS which produced the large cold pool which spreads into the desert. The deformation of the dust plume by the back ground flow can also be seen on 10 June.

2 ms02.gif The initiation and development of the MCS and its cold pool as simulated by WRF. Shown is 1 hr accumulated rainfall (color shading), 10m winds (vectors), 7 m/s 10m isotach (red contour), dust uplift potential (gray shading), and 700 hPa vertical velocities exceeding 3m/s (black dots). The animation runs from the simulation initialization at 0000 UTC 07 June to 0000 UTC 11 June 2010. Note the strong initiation of systems on 07 June over the Hoggar Mountains compared to over the Aïr Mountains and the delay in convective organization when compared to ms01.gif.

3 ms03.gif Cross section through the leading edge of the cold pool as it travels into the desert. Shown is wind (vectors), specific humidity (color contours), and virtual potential temperature (black contours) from 0 to 2 km above sea-level.
