# Supplementary figures and images for: The formation of a large summertime Saharan dust plume: Convective and synoptic-scale analysis
Source: J Geophys Res Atmos. 2014 Feb 26;119(4):1766–85. doi: 10.1002/2013JD020667 (PMC4379907; doi:10.1002/2013JD020667)

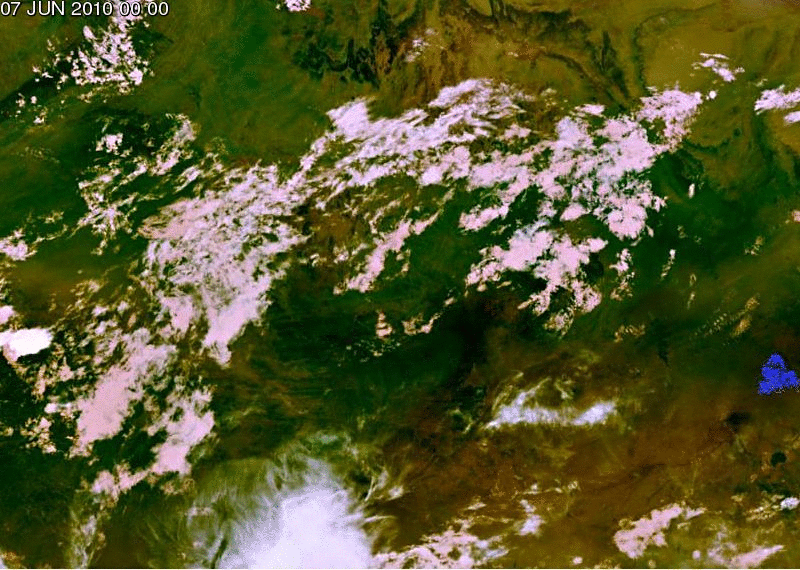

Supplement: Supplementary file 2 — Figure S1 [file jgrd0119-1766-sd2.gif]

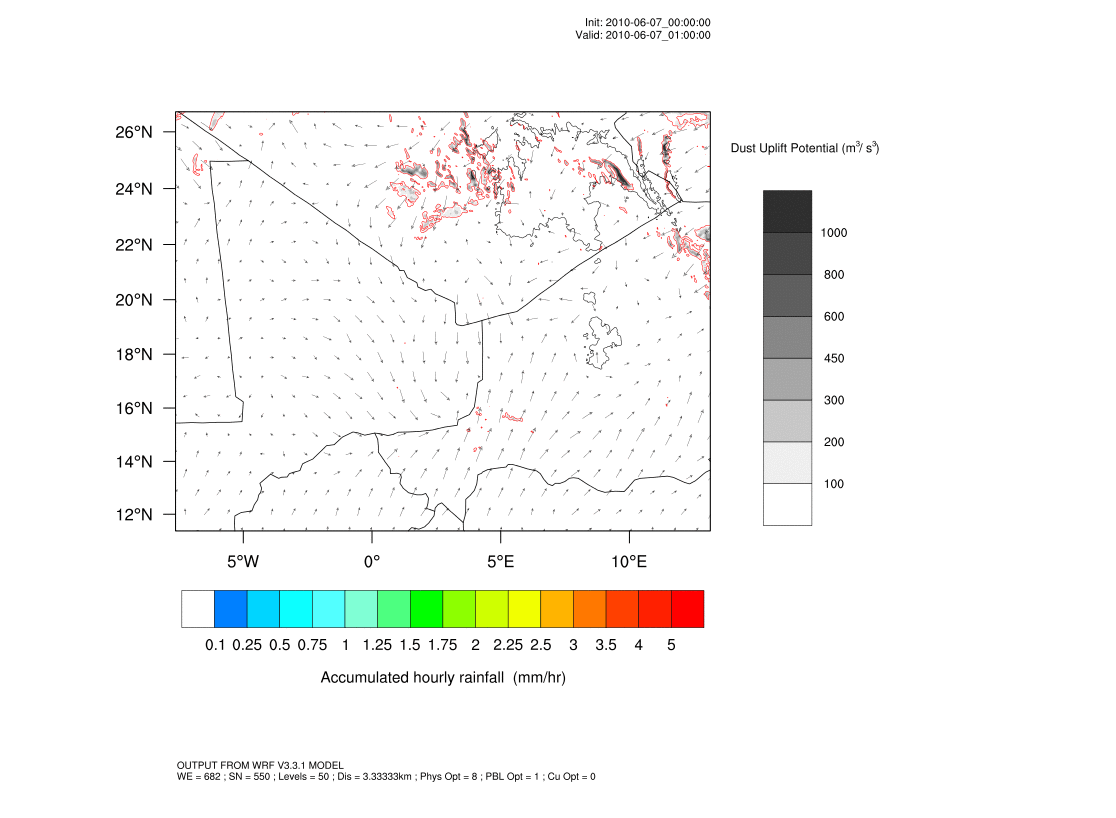

Supplement: Supplementary file 3 — Figure S2 [file jgrd0119-1766-sd3.gif]

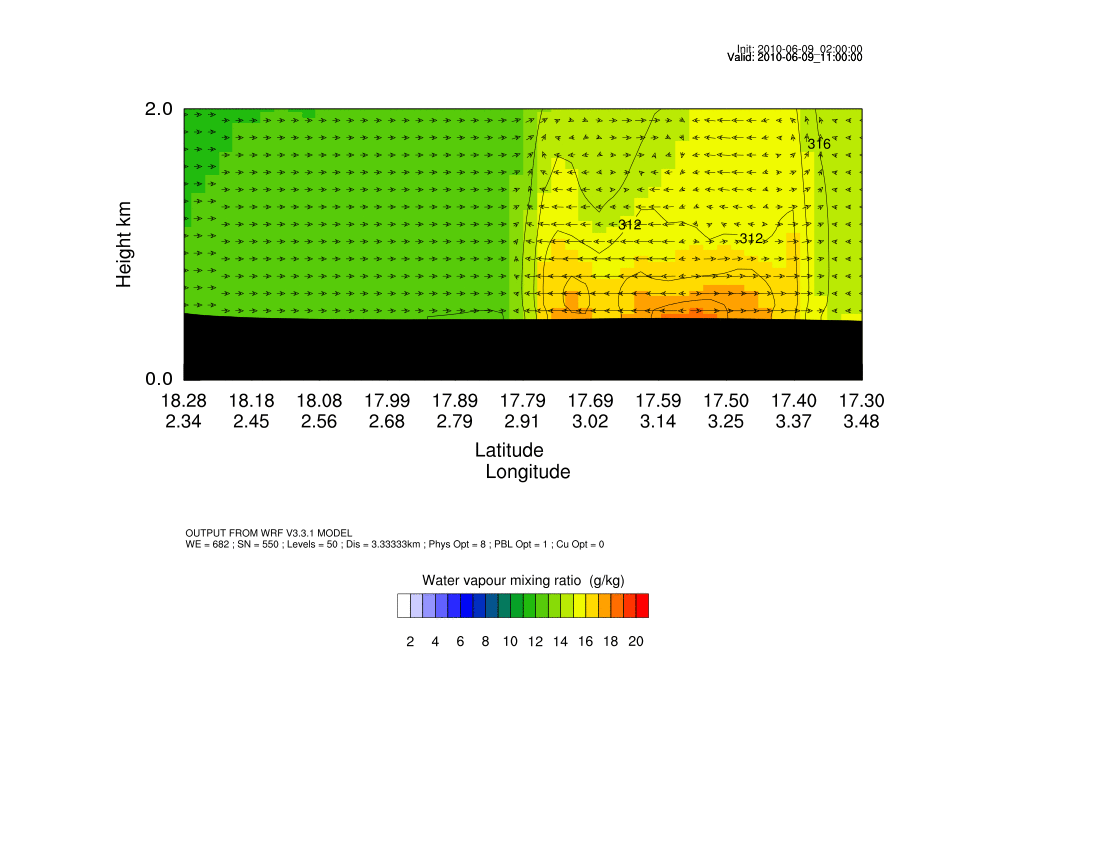

Supplement: Supplementary file 4 — Figure S3 [file jgrd0119-1766-sd4.gif]
